# *Corynebacterium glutamicum* CrtR and Its Orthologs in *Actinobacteria*: Conserved Function and Application as Genetically Encoded Biosensor for Detection of Geranylgeranyl Pyrophosphate

**DOI:** 10.3390/ijms21155482

**Published:** 2020-07-31

**Authors:** Nadja A. Henke, Sophie Austermeier, Isabell L. Grothaus, Susanne Götker, Marcus Persicke, Petra Peters-Wendisch, Volker F. Wendisch

**Affiliations:** 1 Faculty of Biology & CeBiTec, Bielefeld University, 33615 Bielefeld, Germany; n.henke@uni-bielefeld.de (N.A.H.); sophie.austermeier@leibniz-hki.de (S.A.); grothaus@uni-bremen.de (I.L.G.); sgoetker@cebitec.uni-bielefeld.de (S.G.); petra.peters-wendisch@uni-bielefeld.de (P.P.-W.); 2 Department of Microbial Pathogenicity Mechanisms, Leibniz Institute for Natural Product Research and Infection Biology (HKI), 07745 Jena, Germany; 3 Faculty of Production Engineering, Bremen University, 28359 Bremen, Germany; 4 Faculty of CeBiTec, Bielefeld University, 33615 Bielefeld, Germany; marcusp@cebitec.uni-bielefeld.de

**Keywords:** *C. glutamicum*, regulation of carotenogenesis, GGPP, biosensor

## Abstract

Carotenoid biosynthesis in *Corynebacterium* *glutamicum* is controlled by the MarR-type regulator CrtR, which represses transcription of the promoter of the *crt* operon (P*crtE*) and of its own gene (P*crtR*). Geranylgeranyl pyrophosphate (GGPP), and to a lesser extent other isoprenoid pyrophosphates, interfere with the binding of CrtR to its target DNA in vitro, suggesting they act as inducers of carotenoid biosynthesis. CrtR homologs are encoded in the genomes of many other actinobacteria. In order to determine if and to what extent the function of CrtR, as a metabolite-dependent transcriptional repressor of carotenoid biosynthesis genes responding to GGPP, is conserved among *actinobacteria*, five CrtR orthologs were characterized in more detail. EMSA assays showed that the CrtR orthologs from *Corynebacterium* *callunae*, *Acidipropionibacterium* *jensenii*, *Paenarthrobacter* *nicotinovorans*, *Micrococcus* *luteus* and *Pseudarthrobacter* *chlorophenolicus* bound to the intergenic region between their own gene and the divergently oriented gene, and that GGPP inhibited these interactions. In turn, the CrtR protein from *C. glutamicum* bound to DNA regions upstream of the orthologous *crtR* genes that contained a 15 bp DNA sequence motif conserved between the tested bacteria. Moreover, the CrtR orthologs functioned in *C. glutamicum* in vivo at least partially, as they complemented the defects in the pigmentation and expression of a P*crtE*_*gfp*uv transcriptional fusion that were observed in a *crtR* deletion mutant to varying degrees. Subsequently, the utility of the P*crtE*_*gfp*uv transcriptional fusion and chromosomally encoded CrtR from *C. glutamicum* as genetically encoded biosensor for GGPP was studied. Combined FACS and LC-MS analysis demonstrated a correlation between the sensor fluorescent signal and the intracellular GGPP concentration, and allowed us to monitor intracellular GGPP concentrations during growth and differentiate between strains engineered to accumulate GGPP at different concentrations.

## 1. Introduction

Microbial single-cell biosensors have become valuable tools in metabolic engineering due to their easy detection through a fluorescent output signal, their single-cell resolution and their compatibility with viable cells [1,2]. Biosensors facilitate the screening or selection process for a desired product by sensing the presence of the inconspicuous molecule coupled to a conspicuous reporter [2]. These reporters are often based on transcription regulators or riboswitches, as these molecules undergo a conformational change triggered by binding of the analyte, which is directly linked to transcriptional control of the reporter gene [1,2]. Natural biosensors are rare because of the lack of known regulators that are specific for the detection of a desired metabolite. Therefore, intense efforts have been undertaken in biosensor identification and characterization in order to monitor intracellular concentrations of industrially relevant compounds. Rational strain engineering for industrial purposes is often limited by the high complexity of metabolic networks. On the other side, classical strain development based on random mutagenesis is typically limited by the screening capacity, and often lacks an easy to manage readout system to judge the performance of the generated mutants [3]. Biosensors allow high-throughput screening for the monitoring of the production performance, at least for products accumulating intracellularly, while production performance for secreted products can only be deduced indirectly [1,4,5]. Biosensors have been shown to augment and accelerate metabolic engineering based on a new build-test-learn cycle [5].

Isoprenoid pyrophosphates such as GGPP are typically present in low concentrations in the cell. Therefore, an effective biosensor is sought for terpenoid process/strain optimization. Isoprenoid pyrophosphates are building blocks for the synthesis of many high-value terpenoids, including the carotenoid astaxanthin or the sesquiterpenoid patchoulol [6,7,8]. These important secondary metabolites find various applications in the food, feed and cosmetic industries, either as additives or as high-performance ingredients in the health industry [9,10]. Chemical synthesis as well as isolation from natural sources is expensive and/or results in insufficient amounts, and thus the microbial production of terpenoids is receiving increasing attention [11,12]. As an example, the high-value astaxanthin is produced with the microalgae *Haematococcus pluvialis* [13], the red yeast *Xanthophyllomyces dendrorhous* [14] and the bacterium *Paracoccus carotinifaciens* [15]. Microbial carotenoid production by engineered strains is on the rise, as much higher production titers, for example of 6.5 g/L β-carotene with optimized *Yarrowia lipolytica*, can be achieved [16]; however, the industrial production of carotenoids by engineered organisms is currently rare. Besides the intensive engineering of the precursor supply and the optimization of terminal carotenoid biosynthesis, central carbon fluxes as well as cofactor-regeneration might be promising targets [16] in order to achieve carotenoid production titers of >10 g/L. There are two engineered biosensors that exist pertaining to the detection of mevalonate, an intermediate of the mevalonate pathway of isopentenyl pyrophosphate (IPP) biosynthesis [17,18], which cannot be used to monitor IPP biosynthesis via the MEP pathway. Direct IPP sensing has been achieved by a synthetic fusion of the IPP-binding isopentenyl pyrophosphate:dimethylallyl pyrophosphate isomerase Idi with the DNA-binding domain of AraC [19]. This fluorescence reporter responded to the extracellular addition of mevalonate to an *E. coli* strain equipped with the mevalonate pathway; however, no direct evidence was observed that intracellular IPP was sensed by the synthetic biosensor [19]. Such orthogonal biosensors are supposed to interact with the endogenous cellular network less commonly, which might be favorable for scoring production [19], but might be a disadvantage when native regulatory mechanisms are examined.

Here, a biosensor based on the metabolite-dependent MarR-type transcriptional repressor CrtR from *C. glutamicum* is described. This soil bacterium with GRAS status has been used safely in industrial amino acids production for over 60 years [20] since its discovery as a natural glutamate producer [21]. *C. glutamicum* has been metabolically engineered for the sustainable production of various, mostly nitrogenous, compounds [22]. Notably, *C. glutamicum* is a natural carotenoid producer, and its yellow pigmentation is due to the unusual C50 carotenoid decaprenoxanthin [23,24]. The carotenoid precursor GGPP is synthesized from IPP and DMAPP (dimethylallyl pyrophosphate), which are generated from pyruvate and GAP in the MEP pathway [23,24,25], primarily by the prenyltransferase IdsA [26]. The genes that are necessary for the conversion of GGPP to the final yellow decaprenoxanthin are organized in a single operon (*crtE*, *cg0722*, *crtB*, *crtI*, *crtY_e_*, *crtY_f_*, *crtEb)* transcribed from P*crtE* [24]. The knowledge about carotenogenesis in *C. glutamicum* has guided metabolic engineering in such a way as to enhance production of the native decaprenoxanthin [23], and to enable the production of nonnative C40 and C50 carotenoids [27,28,29], including the industrially relevant astaxanthin [30,31]. Several metabolic engineering approaches have been used to improve the production of terpenoids by *C. glutamicum*. First, *dxs* encoding the committed enzyme in the MEP pathway was overexpressed [32,33]. Second, balancing the DMAPP to IPP ratio via the overexpression of *idi* improved patchoulol production when combined with *dxs* overexpression [8]. Third, the overproduction of the two endogenous GGPP synthases IdsA and CrtE enhanced decaprenoxanthin production due to increased synthesis of GGPP [26]. The overexpression of these genes from IPTG-inducible promoters was orthogonal and independent from endogenous transcription regulatory feedback. Recently, a membrane-fusion protein comprising CrtZ and CrtW was published, and it was shown that the additional overexpression of precursor biosynthesis genes enhanced astaxanthin product formation [31]. In this regard, precursor-dependent transcriptional regulation may be beneficial, for example in the on-demand conversion of GGPP to the chosen target terpenoid. A biosensor system for the detection of GGPP would represent a powerful tool for strain development, in particular with regard to investigations into the MEP pathway concerning efficient precursor supply. *C. glutamicum* possesses the transcriptional repressor CrtR for the control of decaprenoxanthin biosynthesis [23]. Like most MarR-type regulators, *C. glutamicum* CrtR represses gene transcription by binding to the intergenic region between its own gene and the divergently oriented *crt* operon [23,34]. In vitro analysis showed that isoprenoid pyrophosphates act as inducers of CrtR in *C. glutamicum*. CrtR binding to P*crtE* was inhibited by GGPP, and to lesser extents by FPP, GPP, DMAPP and IPP [23]. Thus, in *C. glutamicum*, GGPP leads to the derepression of the *crt* operon and its own gene by CrtR in a metabolite-dependent feed forward mechanism [23].

CrtR has also been associated with the light-dependent regulation of carotenogenesis, although the mechanism remains unclear [35]. CrtR homologs are found mainly in actinobacteria, and *crtR* genes often cluster with carotenoid biosynthetic genes and/or a *mmpL*-like transporter gene [23]. This suggested a conserved regulatory function of the CrtR orthologs with respect to transcriptional control of carotenoid biosynthetic genes and/or a *mmpL*-like transporter gene in other actinobacteria. MmpL (mycobacterial membrane protein large) proteins often export hydrophobic or lipid-like substances across the cell membrane in mycobacteria [36]. Here, we studied if, and to what extent, the function of CrtR as a metabolite-dependent transcriptional repressor of carotenoid biosynthesis genes responding to GGPP is conserved among actinobacteria. Moreover, we developed the first genetically encoded biosensor system for the detection of intracellular GGPP based on CrtR from *C. glutamicum*.

## 2. Results

### 2.1. CrtR Orthologs from Actinobacteria Showed Binding to Their Own Promoters and Derepression by GGPP

**Figure 1:**
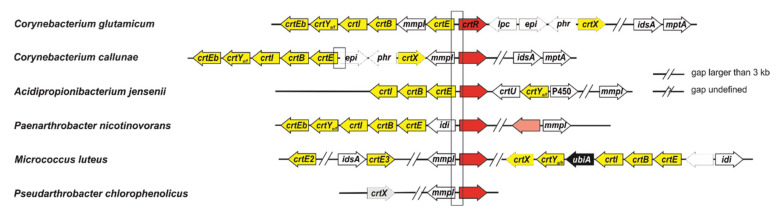
Genomic organization of *crtR* from *C. glutamicum* ATCC 13,032 and the *crtR* orthologs from *C. callunae* DSM 20,147, *A. jensenii* DSM 20,535, *P. nicotinovorans* Hce-1, *M. luteus* NCTC 2665 and *P. chlorophenolicus* A6. Boxed areas highlight the putative promoter regions tested in bandshift assays. The *crtR* orthologs (given in red) are transcribed divergently either to *crt* genes (given in yellow) or to *mmpL* genes. *P. nicotinovorans* contains in addition a *crtR* paralog (given in pink), which is transcribed divergently to gene *mmpL*. Other carotenoid associated genes (given in white) and genes for prenylation (given in black) in the close vicinity of *crtR* genes are included.

Previously, the MarR-type regulator CrtR was identified as a GGPP-dependent repressor of the carotenogenic gene cluster *crtE-mmpl-crtBIY_e/f_Eb*, and was shown to auto-regulate its own expression [23]. Moreover, 94 CrtR homologs with at least a 25% amino acid identity with CrtR from *C. glutamicum* were identified [23]. In order to study if the function of these MarR-type regulators as GGPP-dependent transcriptional repressors of carotenogenesis is conserved, the orthologs of five actinobacteria, with increasing phylogenetic distances between themselves and *C. glutamicum*, were selected for further analysis. The *crtR* genes from *C. callunae*, *A. jensenii* and *P. nicotinovorans*, as well as *C. glutamicum*, have in common that they are co-localized with genes of carotenoid biosynthesis [23] (Figure 1). The phylogenetically more distant *crtR* genes from *P. chlorophenolicus* and *M. luteus* co-localize only with a *mmpL* gene. The genome of *P. chlorophenolicus* lacks carotenogenesis genes except *crtR*, whereas the carotenogenic genes of *M. luteus* are encoded in loci distant from *crtR* (Figure 1).

**Figure 2:**
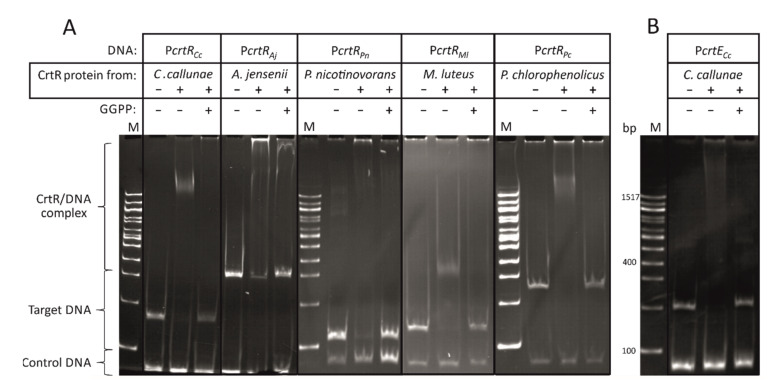
In vitro characterization of CrtR orthologs. (**A**) Bandshift assays of CrtR orthologs from *C. callunae*, *A. jensenii*, *P. nicotinovorans*, *M. luteus* and *P. chlorophenolicus* with their respective own putative promoter region and the inhibition of the binding by GGPP. (**B**) Bandshift assays of CrtR from *C. callunae* with a putative *crtE* promoter region and the inhibition of the binding by GGPP. The presense or absence of CrtR protein and GGPP are indicated by “+” and “−“, respectively.

The CrtR orthologs from *C. callunae* (CrtR*_Cc_*), *A. jensenii* (CrtR*_Aj_*), *P. nicotinovorans* (CrtR*_Pn_*), *P. chlorophenolicus* (CrtR*_Pc_*) and *M. luteus* (CrtR*_Ml_*) were fused with an N-terminal His-tag, and the proteins were purified by Ni-NTA affinity chromatography. In order to test if *crtR* autoregulation—as observed for CrtR from *C. glutamicum—*is conserved, each protein was tested for binding to the intergenic DNA sequences between its own gene and the divergently transcribed gene (Figure 2). Indeed, all CrtR orthologs analyzed bound to the DNA sequences upstream of their own *crtR* gene (Figure 2). This indicated *crtR* autoregulation and/or regulation of the respective divergently transcribed genes (*mmpl* for *C. callunae, M. luteus* and *P. chlorophenolicus*, *crtE* for *A. jensenii* and *idi* for *P. nicotinovorans*) (Figure 1). The finding that *A. jensenii* CrtR, *P. nicotinovorans* CrtR and *M. luteus* CrtR did not shift all target DNA may either indicate that the CrtR protein–target DNA interaction is less tight in these bacteria, or it may be due to technical reasons, e.g., due to the purification of the tagged proteins that may differ between the five CrtR proteins analyzed.

Since GGPP inhibits the binding of CrtR from *C. glutamicum* to its target promoter [23], it was tested if GGPP could also inhibit the binding of the other CrtR orthologs to their respective target DNA. Indeed, GGPP inhibited the interaction between the tested CrtR proteins and their target DNA (Figure 2A).

Thus, these in vitro results revealed that the binding of CrtR orthologs, from several actinobacteria, to their own upstream DNA sequences, and the inhibitory effect of GGPP, are conserved.

For *C. callunae*, a second putative target DNA sequence was tested, namely the intergenic region between its carotenogenic gene cluster *crtEBIY_e/f_Eb*, which is located a few genes upstream of *crtR* in this bacterium, and the divergently transcribed gene *epi* (Figure 1). This intergenic DNA sequence from *C. callunae* was bound by a CrtR protein from *C. callunae* (CrtR*_Cc_*) unless GGPP was added (Figure 2B). Thus, the GGPP-dependent regulation of carotenoid biosynthesis genes by CrtR is conserved at least in the closely related *C. callunae*, and possibly in other actinobacteria.

### 2.2. CrtR from C. glutamicum Binds to Heterologous crtR Promoter DNA Sequences

**Figure 3:**
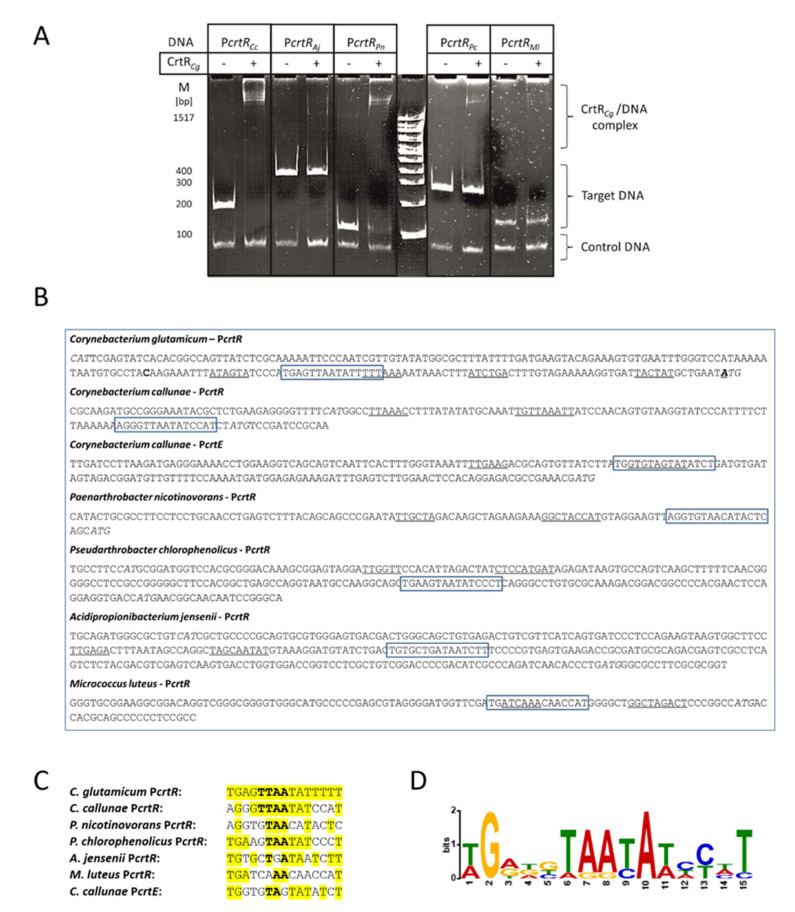
Characterization of CrtR from *C. glutamicum* in vitro. (**A**) Bandshift assays of His-tagged CrtR protein from *C. glutamicum* (CrtR_Cg_) and the intergenic DNA sequences between the *crtR* orthologs from *C. callunae*, *A. jensenii*, *P. nicotinovorans*, *M. luteus* and *P. chlorophenolicus,* and the respective divergently transcribed genes. (**B**) Putative -10 and -35 promoter DNA sequences (underlined), translation start codons (italics) and the putative conserved CrtR binding sequences (boxed). The mapped transcriptional start sites of *C. glutamicum crtR* and *crtE* are given in bold. (**C**) Putative conserved CrtR binding sequences (conserved nucleotides are given in yellow; the **TTAA** sequence that was shown previously to be required for *C. glutamicum* CrtR binding by mutational analysis is depicted in bold face). (**D**) The graphical representation of the derived consensus DNA binding motif of the CrtR proteins from *C. glutamicum*, *C. callunae*, *A. jensenii*, *P. nicotinovorans*, *M. luteus* and *P. chlorophenolicus* (designed using WebLogo).

The binding of the *C. glutamicum* CrtR protein to heterologous *crtR* promoter DNA sequences was studied in order to (i) test if this specific DNA binding is conserved across the actinobacteria analyzed, and, if it is, to (ii) identify the putative DNA sequence motif. The intergenic DNA sequences between the five orthologous *crtR* genes and the respective divergently transcribed genes were used in a bandshift assay with His-tagged CrtR proteins from *C. glutamicum* (Figure 3A). The strong binding of CrtR*_Cg_* to the *crtR* promoter sequences from *C. callunae* and *P. nicotinovorans* was detected, whereas the interactions between CrtR*_Cg_* and the *crtR* promoter sequences from *A. jensenii*, *M. luteus* and *P. chlorophenolicus* were weak (Figure 3A).

Previously, we narrowed down the target DNA sequence, to which CrtR from *C. glutamicum* binds, to 19 bp (5′-CCCATGAG**AATT**TATTTTT-3′), and mutational analysis revealed that exchanging the central four nucleotides (**TTAA**) simultaneously interfered with binding [23]. Inspection of the DNA sequences upstream of the *crtR* genes from *C. callunae*, *A. jensenii*, *P. nicotinovorans*, *M. luteus* and *P. chlorophenolicus* revealed that this motif was present in the intergenic DNA regions in these species (Figure 3B), and conserved to some extent (Figure 3C). It is evident that conservation of the central **TTAA** sequence is not sufficient to explain the observed binding preferences of CrtR*_Cg_* and the variuos *crtR* promoter sequences studied. It remains to be elucidated how specific nucleotides of the 15 bp motif (other than the central **TTAA**) affect the binding of CrtR*_Cg_* protein to DNA.

The derived consensus DNA binding motif of CrtR proteins from the studied species is depicted in Figure 3D, with sequence conservation and relative frequency for each nucleotide position.

### 2.3. CrtR Orthologs from Actinobacteria Affected Carotenogenesis and Expression of a CrtE Transcriptional Fusion in C. glutamicum In Vivo

**Figure 4:**
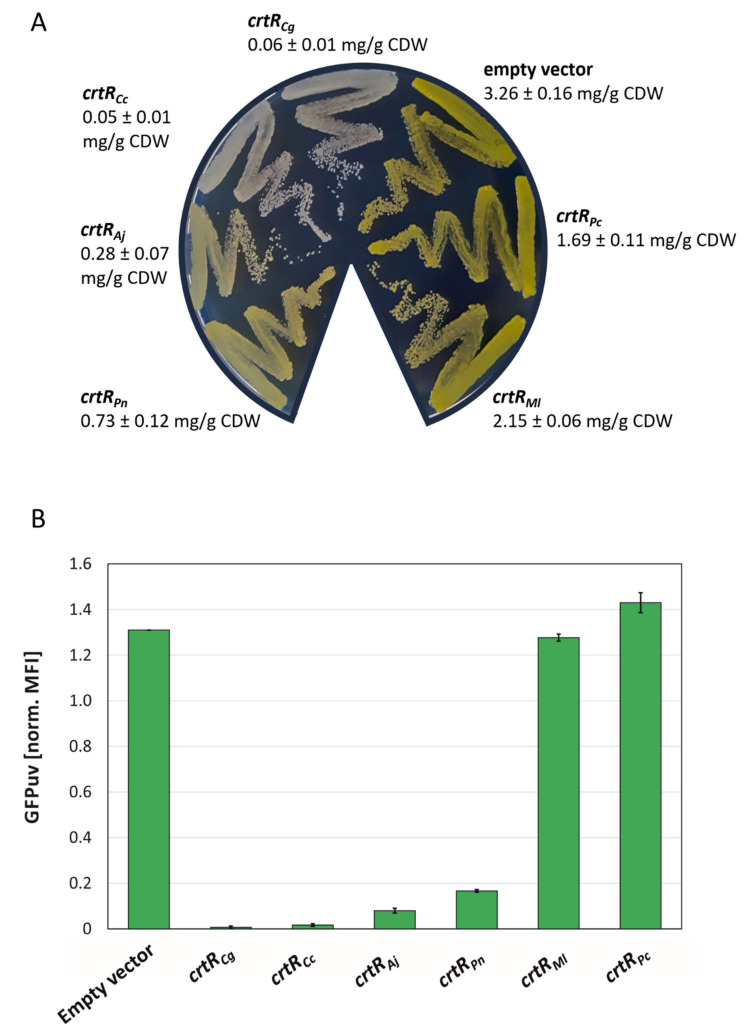
In vivo characterization of CrtR orthologs in *C. glutamicum* WTΔ*crtR*. (**A**) Phenotypes on LB plates after incubation at 30 °C for 24 h and carotenoid concentration in mg/g CDW (β-carotene equivalents) of WTΔ*crtR* strains harboring pTEST derivatives expressing *crtR* genes from the indicated bacteria. (**B**) Flow cytometry analysis of the strains depicted in (A) during exponential growth in LB. Mean fluorescence intensities (MFI) of GFPuv signals were normalized to autofluorescence and shown for at least two biological replicates.

To monitor the promoter activity of the carotenogenic gene cluster of *C. glutamicum* in vivo, the promoter probe vector pEPR1 was used [37]. This reporter system was employed to determine the promoter activity of the carotenogenic promoter (P*crtE*) from *C. glutamicum* in the absence of endogenous chromosomally encoded CrtR, but in the presence of CrtR orthologs from other actinobacteria. The CrtR orthologs were at different phylogenetic distances compared to CrtR from *C. glutamicum,* and therefore different protein identities: *C. callunae* (62% identity), *A. jensenii* (57% identity), *P. nicotinovorans* (53% identity), *M. luteus* (35% identity) and *P. chlorophenolicus* (35% identity). To this end, the *crtR* orthologs from *C. callunae*, *A. jensenii*, *P. nicotinovorans, M. luteus* and *P. chlorophenolicus*, as well as the *crtR* from *C. glutamicum* as reference, were expressed from the strong, constitutive promoter P*gap* in divergent orientation to the P*crtE*_*gfp*uv transcriptional fusion. The respective vectors were named pTEST_CrtR*_Cg_*, pTEST_CrtR*_Cc_*, pTEST_CrtR*_Aj_*, pTEST_CrtR*_Pn_*, pTEST_CrtR*_Ml_* and pTEST_CrtR*_Pc_* (Figure S1). First, maximal repression using the vector pTEST-*crtR_Cg_* was determined and compared to a two-vector system, in which the expression of *crtR* and the reporter gene fusion P*crtE_gfp*uv were decoupled; *crtR* was expressed by IPTG-inducible pEC-XT_*crtR_Cg_*, while pEPR1_P*crtE* contained the reporter gene fusion P*crtE_gfp*uv (Figure S1). In the strains carrying the P*crtE_gfp*uv fusion, but lacking *crtR* (WTΔ*crtR*(pTEST) and WTΔ*crtR*(pEPR1_P*crtE*), pigmentation due to decaprenoxanthin accumulation (3.2–3.3 mg/g CDW) and GFPuv fluorescence (1.3 normalized MFI) were high (Figure S2). The IPTG-inducible expression of *crtR* in the two-vector system reduced decaprenoxanthin accumulation and GFPuv fluorescence 38- and 32-fold, respectively (Figure S2). Reduction of decaprenoxanthin accumulation and GFPuv fluorescence using pTEST_*crtR_Cg_* (200- and 55-fold, respectively; Figure S2) was even higher (*crtR_Cg_* is transcribed from the strong and constitutive P*gap*, shown above). Next, the in vivo effects of the CrtR orthologs were tested. Constitutive overexpression of the *crtR* orthologs from the *gap* promoter revealed differential effects on carotenogenesis and expression of the P*crtE*_*gfp*uv transcriptional fusion (Figure 4). WTΔ*crtR* carrying the empty vector pTEST showed the expected intense yellow pigmentation due to derepression of the chromosomal carotenoid biosynthesis genes (Figure 4A), as well as the derepressed expression of the P*crtE*_*gfp*uv transcriptional fusion (Figure 4B). Upon plasmid-borne expression of CrtR repressor genes from *C. glutamicum*, *C. callunae*, *A. jensenii* and *P. nicotinovorans*, pigmentation was strongly reduced to less than 1 mg/g CDW (Figure 4A), which corresponded to the strongly reduced expression of the P*crtE*_*gfp*uv transcriptional fusion (Figure 4B). Repression by CrtR from *C. callunae* was nearly as tight as that by endogenous CrtR, leading to a relative GFPuv signal of less than 0.1 (Figure 4B). CrtR orthologs from *A. jensenii* and *P. nicotinorovans* also repressed the *crtE* promoter fusion very efficiently, resulting in a GFPuv signal of less than 0.2 (Figure 4B). Upon expression of *M. luteus crtR*, carotenoid biosynthesis was reduced to a much lesser extent (Figure 4A), while the expression of the P*crtE*_*gfp*uv transcriptional fusion was as high as in the empty vector control (Figure 4B). CrtR from *P. chlorophenolicus* reduced pigmentation, but expression of the P*crtE*_*gfp*uv transcriptional fusion was unaffected (Figure 4A,B).

Taken together, the repression of carotenogenesis, and of the expression of *crtE* transcriptional fusion, in *C. glutamicum* in vivo by CrtR orthologs from other actinobacteria was possible. The efficacy was highest for closely related species, and it decreased with phylogenetic distance.

### 2.4. Construction and Analysis of a GGPP Biosensor

**Figure 5:**
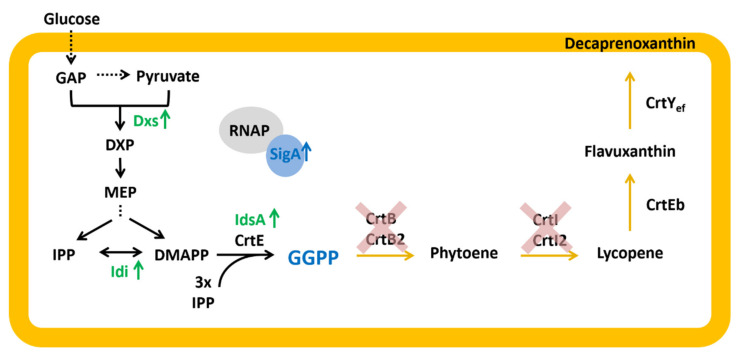
GGPP and decaprenoxanthin biosynthesis pathway in *C. glutamicum*. GAP: glyceraldehyde 3-phosphate, DXP: 1-deoxy-1-xylulose-5-phosphate; MEP: methylerythritol phosphate; IPP: isopentenyl pyrophosphate; DMAPP: dimethylallyl pyrophosphate; GGPP: geranylgeranyl pyrophosphate; RNAP: RNA-Polymerase core enzyme; SigA: housekeeping primary sigma factor A; Dxs: 1-deoxy-1-xylulose5-phosphate synthase; Idi: isopentenyl pyrophosphate isomerase; IdsA/CrtE: GGPP synthase; CrtB/CrtB2: phytoene synthase; CrtI/I2: phytoene desaturase; CrtEb: lycopene elongase; CrtY_ef_: C50 ε-cyclase; genes overexpressed in strains GGPPA and GGPPB are shown in green, genes deleted on both strains are indicated by red crosses; blue shows genes overexpressed only in strain GGPPB.

The expression control of P*crtE*_*gfp*uv transcriptional fusion by CrtR from *C. glutamicum* has been described previously [19], and the results from above prompted us to consider its use in combination with chromosomally encoded *crtR* as a GGPP biosensor, i.e., using *C. glutamicum* WT(pEPR1_P*crtE)* as a biosensor strain. Targeted metabolic engineering typically addresses four modules: carotenoid biosynthesis, precursor supply, central carbon metabolism and redox cofactor regeneration. A CrtR-based biosensor may allow us to simultaneously optimize the latter three modules via fluorescence reporter output. While GGPP inhibits the DNA binding of CrtR [23], no feeding regimen is known to predictably alter the intracellular GGPP concentration. Therefore, a genetic approach was chosen, and two *C. glutamicum* strains, expected to accumulate GGPP to intracellular concentrations different from the wild type, were constructed (Figure 5).

First, the conversion of GGPP by the endogenous phytoene synthases CrtB [24] was prevented through the deletion of *crtB* and *crtB2I’I2* from the chromosome of the *C. glutamicum* WT yielding strain WTΔ*crtB*Δ*crtB2I’I2* (Table 1). Second, the supply of the precursor molecules DMAPP and IPP was increased by overexpression of the genes encoding the MEP pathway enzymes 1-deoxy-D-xylulose 5-phosphate synthase (Dxs) and Idi. It is known for *C. glutamicum* [28] and other organisms that the first enzymatic step in the MEP pathway strongly limits the flux [38,39,40]. Dxs is supposed to be feedback-regulated by isoprenoid pyrophosphates [38], and the overexpression of *dxs* was shown to increase the flux towards terpenoid biosynthesis [28,30,33]. In addition, the ratio of DMAPP to IPP was shown to be important for optimized isoprenoid production, and *idi* overexpression equilibrates intracellular concentrations of DMAPP and IPP [28,33,39]. Third, GGPP synthesis was improved by plasmid-driven overexpression of the major GGPP synthase gene *idsA* from *C. glutamicum* [26] (pEC-XT_*idsA*; Table 1). Combining the three strategies resulted in strain GGPPA (WTΔ*crtB*Δ*crtB2I’I2* (pEKEx3-*dxs_idi*) (pECXT-*idsA*)) (Table 1) (Figure 5). Since engineering of the RNA polymerase sigma factor A improved isoprenoid carotenoid production [41], *sigA* from *C. glutamicum* was overexpressed in a synthetic operon with *idsA* (pEC-XT_*idsA_sigA*) (Table 1). The resulting strain GGPPB (WTΔ*crtB*Δ*crtB2I’I2* (pEKEx3-*dxs_idi*) (pECXT-*idsA*_*sigA*)) differs from strain GGPPA only by the additional overexpression of *sigA* (Table 1) (Figure 5). The intracellular GGPP concentrations of the *C. glutamicum* strains WT, GGPPA and GGPPB were expected to differ.

**Figure 6:**
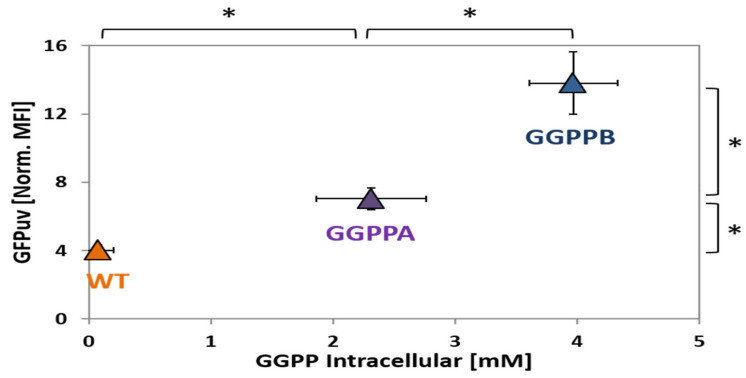
Biosensor-based differentiation between strains accumulating different GGPP concentrations. The intracellular GGPP concentrations are given in mM, and the GFPuv signals in mean fluorescence intensities were normalized to autofluorescence. Strains WT (pEPR1_P*crtE*), GGPPA (pEPR1_P*crtE*) and GGPPB (pEPR1_P*crtE*) were cultivated in CGXII (100 mM Gluc + 100 µM IPTG) and data were taken after 12 h. Statistical significance was calculated with paired Student t-test (two-tailed); *: *p*-value < 0.05.

After transformation with the biosensor plasmid pEPR1_P*crtE*, the strains were grown in CGXII minimal medium with glucose. Samples were taken during exponential growth 12 h after inoculation and analyzed by LC-MS (Figure 6). As expected, WT (pEPR1_P*crtE*) accumulated the lowest GGPP concentration, with less than 0.1 mM GGPP (Figure 6). In comparison, GGPPA (pEPR1_P*crtE*) accumulated about 23-fold more GGPP (2.3 ± 0.5 mM; Figure 6). With 4.0 ± 0.4 mM, strain GGPPB (pEPR1_P*crtE*) exhibited the highest concentration of GGPP, i.e., about 1.7-fold higher than GGPPA (pEPR1_P*crtE*). Thus, it was confirmed that the genetic approach altered the intracellular GGPP concentrations as anticipated.

Since the strains harbored plasmid pEPR1_P*crtE*, the sensing of intracellular GGPP by chromosomally encoded CrtR could be tested. The normalized mean fluorescence intensity (MFI) of the P*crtE_gfp*uv fusion observed after 12 h growth in glucose minimal medium was lowest for WT (pEPR1_P*crtE*) (MFI of 4.0 ± 0.3). The biosensor signal of GGPPB (pEPR1_P*crtE*), of 14.0 ± 1.8, was about two-fold higher than that of GGPPA (pEPR1_P*crtE*) (MFI of 7.0 ± 0.7; Figure 6). Thus, biosensor signal output correlated with intracellular GGPP concentration.

**Figure 7:**
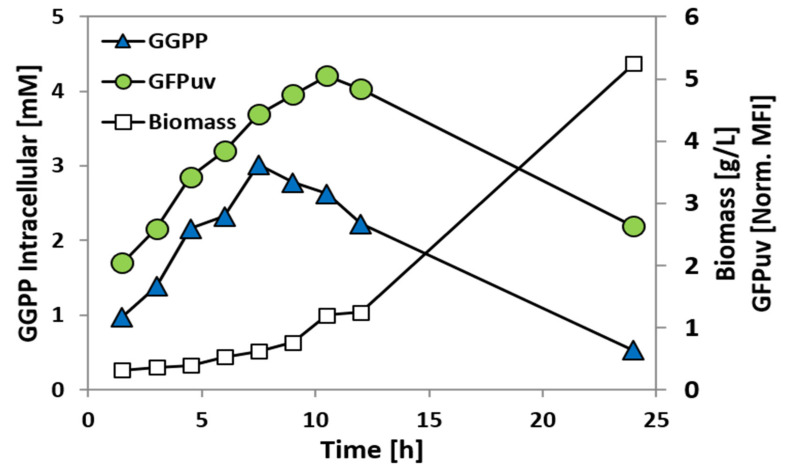
GGPP concentration and GFPuv fluorescence during growth of GGPP accumulating *C. glutamicum* strain GGPPB (pEPR1_P*crtE*). Intracellular GGPP concentration (blue triangles; in mM) and GFPuv signal (green circles; mean fluorescence intensities normalized to autofluorescence) were monitored during growth in CGXII (100 mM Gluc + 100 µM IPTG). Biomass concentrations are given in gCDW/L (empty squares).

In order to determine if the P*crtE_gfp*uv fusion can be used to monitor variations in the GGPP concentration during growth, strain GGPPB (pEPR1_P*crtE*) was analyzed in a time-course experiment in CGXII minimal medium supplemented with 100 mM glucose and 100 µM IPTG (Figure 7). Samples were taken every 1.5 h for determination of the intracellular GGPP concentration and flow cytometry analysis (Figure 7). Both the intracellular GGPP concentration and the GFPuv signal strongly increased in the first 7.5–10.5 h after inoculation. An offset between the GGPP concentration and the GFPuv signal was observed, with the increase in the latter being delayed by about 4 h (Figure 7). This offset may be explained by the time required to synthesize GFPuv after CrtR has sensed an increased GGPP concentration and P*crtE_gfp*uv has been derepressed. The intracellular GGPP concentration reached its maximum approximately after 7.5 h, with about 3 mM GGPP, and decreased afterwards, while the GFPuv reached its maximum after 10.5 h of cultivation (Figure 7).

## 3. Discussion

This study revealed the conserved functions of CrtR orthologs in six actinobacteria with respect to GGPP-dependent regulation. The repression of carotenogenesis, and of the expression of a *crtE* transcriptional fusion, in *C. glutamicum* in vivo was highest for closely related species, and it decreased with phylogenetic distance. The P*crtE*_*gfp*uv transcriptional fusion was suitable for monitoring intracellular GGPP concentrations in a strain with chromosomally encoded *crtR* as a genetically encoded biosensor system.

The conserved role of CrtR orthologs as GGPP-dependent transcriptional regulators suggested that they are relevant for the control of carotenogenesis and/or *mmpL* genes. Although not tested, it is tempting to speculate that these proteins may be used to monitor intracellular GGPP concentrations in their native hosts. *C. glutamicum* and *C. callunae* are close relatives, synthesizing the C50 carotenoid decaprenoxanthin and its glycosides as pigments [25,27]. *P. nicotinovorans* is pigmented most probably due to the accumulation of carotenoids [35]. *M. luteus* is a yellow-pigmented bacterium due to the accumulation of the C50 carotenoid sarcinaxanthin and its glycosides [53]. By contrast, *A. jensenii* does not synthesize a carotenoid, but the polyene pigment granadaene [54], while *P. chlorophenolicus* is a non-pigmented soil bacterium [55]. Interestingly, relatives of *P. chlorophenolicus*, such as *Arthrobacter arilaitensis*, are pigmented most probably due to the accumulation of C50 carotenoids, and are found on the surface of smear-ripened cheeses [55,56]. Thus, carotenogenesis is not conserved in actinobacteria possessing CrtR orthologs that control the expression of their own gene and/or the divergently oriented gene(s) in a GGPP-dependent manner. Besides carotenogenesis genes, *mmpL* genes are also transcribed divergently to *crtR*, and are presumably controlled by CrtR. MmpL transporters are considered candidate targets for the development of anti-tuberculosis drugs [57], as they couple lipid synthesis and the export of bulky, hydrophobic substrates [58]. Thus, MmpL proteins are essential for the cell envelope, and support the infectivity and persistence of *M. tuberculosis* in its host [59]. Moreover, MmpL proteins of *M. tuberculosis* are involved in the oxidative stress response [60]. 

MarR-type transcriptional regulators, including CrtR and its orthologs, are found in all bacteria, and are natural sensors that allow adaptation to environmental stresses such as ROS, toxic compounds or antibiotics (hence the name multiple antibiotic resistance regulators) [61]. As shown for the CrtR orthologs studied here (see Figure 1 and Figure 2), MarR-type regulators typically repress genes in the close vicinity [34]. MarR-type regulators typically bind and respond to low-molecular-weight compounds [34,62], such as GGPP for the CrtR orthologs. *C. glutamicum* possesses nine MarR-type regulators, eight of which have been characterized in some detail. RosR [63], CosR [64], OhsR [65] and OsmC [66] play roles in the response to ROS stress, whereas CarR [67], MalR [68] and PhdR [69] deal with other environmental stresses, such as toxic compounds or cell-membrane associated stress. The GGPP-dependent control of carotenogenesis by CrtR from *C. glutamicum* and its orthologs studied here may be considered a stress response, since the antioxidative properties of carotenoids counteract oxidative stress. This function would be in line with the CrtR-mediated control of *mmpL* genes that are involved in the oxidative stress response (see above).

MarR-type regulators typically bind to a palindromic 16–20 bp target site that overlaps with the -10 or -35 promoter regions for steric inhibition of RNA polymerase binding [34]. Previously, a 19 bp DNA sequence with a central TTAA motif was shown to be essential for the binding of CrtR from *C. glutamium* to its DNA target site [23]. Here, we showed that CrtR from *C. glutamicum* bound to DNA sequences upstream of *crtR* from other actinobacteria, and inspection of the DNA sequences revealed a conserved 15 bp binding motif, including the central TTAA base pairs (see Figure 3). The binding of CrtR*_Cg_* decreased with increasing deviation from the consensus motif (see Figure 3). The CrtR binding motif is typical for the MarR-type family of transcriptional regulators [34]. The observed graded effect of CrtR*_Cg_* binding to promoter sequences with increasing deviation from the consensus motif in vitro was congruent with the in vivo finding that different CrtR orthologs affected the pigmentation and expression of the P*crtE*_*gfp*uv transcriptional fusion more weakly when their phylogenetic distance to *C. glutamicum* was greater (see Figure 4). This is in line with the phylogenetic analysis of CrtR orthologs [23].

The application of biosensors has become a prominent tool in strain development over the last few years [5,70]. In this study, CrtR from *C. glutamicum* was demonstrated to be the first genetically encoded biosensor for the detection of GGPP that allows one to distinguish between *C. glutamicum* strains that have accumulated GGPP to different intracellular concentrations (Figure 6), and to monitor GGPP accumulation over time during growth (Figure 7). Since the CrtR-based biosensor system was suitable for the detection of intracellular GGPP concentrations between 0.1 and at least 4 mM (Figure 6), it is plausible that the described system is applicable to the screening of mutants accumulating GGPP well above wild type levels, and their enrichment/isolation, by flow cytometry. As an alternative application, the on-demand expression control of GGPP converting enzymes in response to intracellular GGPP concentration can be envisioned for strain optimization with respect to the production of GGPP-derived diterpenoids and/or carotenoids. On-demand production may be established by transcriptional fusion of the P*crtE* to the gene of interest, e.g., a diterpenoid synthase. This approach may improve production as the terminal biosynthesis pathway is initiated only in the presence of high concentrations of the precursor GGPP, which may prevent the accumulation of toxic GGPP concentrations. Biosensor approaches to on-demand expression control have been successfully applied, e.g., to improve lysine production by *C. glutamicum* [71,72].

The central role of GGPP as a terpenoid and carotenoid precursor suggests a wide application range for the GGPP-based biosensor developed here [10], since the tens of thousands of terpenoids derived from GGPP represent one of the biggest sources of valuable natural products for human use [73].

## 4. Materials and Methods 

### 4.1. Bacterial Strains, Media and Growth Conditions

Strains and plasmids that were used in this study are listed in Table 1. *C. glutamicum* ATCC 13,032 [45] served as the wild type and was used as the basic strain for genetic engineering. Modifications aimed at higher production levels of GGPP and the establishment of the biosensor system. Precultures of *C. glutamicum* were performed in LB/BHI medium with 50 mM glucose as carbon and energy source [74] supplemented with the appropriate antibiotic at 30 °C and 120 rpm. The main cultures of *C. glutamicum* consisted of 50 mL CGXII medium with 100 mM glucose and 100 µM IPTG and were inoculated to an initial optical density (OD_600_) of 1. The OD_600_ of the cultures was measured with the Shimadzu UV-1202 spectrophotometer (Duisburg, Germany). 

### 4.2. Recombinant DNA Work and Gene Expression

Cloning of plasmids was done in *E. coli* DH5α using PCR-generated fragments that were purified using the NucleoSpin kit (Macherey-Nagel, Düren, Germany). Oligonucleotides were ordered from Metabion GmbH (Planegg/Steinkirchen, Germany) (Table 2). For plasmid construction standard PCR, restriction and dephosphorylation reactions [75] were performed as well as Gibson Assembly [76]. Transformation of *E. coli* was performed via the RbCl method [42]. Cloned DNA insert fragments were verified by sequencing. Transformation of *C. glutamicum* was performed via electroporation using a Gene Pulser Xcell™ (Bio-Rad Laboratories GmbH, Munich, Germany) at 2.5 kV, 200 Ω and 25 µF [74]. For expression of CrtR in the pTEST vector (NA, Table 1 and Table 2) and the production of His-tagged CrtR from various organisms in the pET16b vector (HN, Table 1 and Table 2), the respective genes were amplified from chromosomal DNA using primer pairs NA25/26 and HN83/HN84 for *C. glutamicum*, NA27/28 and HN85/HN86 for *C. callunae*, NA31/32 and HN87/HN88 for *A. jensenii*, NA33/34 and HN89/HN90 for *P. nicotinovorans*, NA39/40 and HN93/HN94 for *M. luteus* and NA41/42 and HN95/HN96 for *P. chlorophenolicus*, respectively. The purified PCR products were cloned into pTEST restricted with *BamHI* and pET16b restricted with *Nde*I using Gibson assembly [76], respectively. Chromosomal DNA was extracted from DSMZ (see Table 1). 

### 4.3. Extraction of Carotenoids from Bacterial Cells and HPLC Analysis

The carotenoid extraction from *C. glutamicum* was performed as described previously [30] using 1 mL of the cell cultures. Pigments were isolated from the cell pellets with a methanol:acetone mixture (7:3) at 60 °C for 15 min with shaking at 500 rpm. The clear supernatant was used for HPLC analysis after centrifugation of the extract for 10 min at 13,000× *g*. The carotenoid concentration of cell extracts was determined through absorbance at 471 nm by high performance liquid chromatography (HPLC) analysis, performed on an Agilent 1200 series HPLC system (Agilent Technologies Sales & Services GmbH & Co. KG, Waldbronn, Germany), including a diode array detector (DAD) for UV/visible (Vis) spectrum recording. Separation of the carotenoids was performed by application of a column system consisting of a precolumn (LiChrospher 100 RP18 EC-5, 40 × 4 mm, CS-Chromatographie, Langerwehe, Germany) and a main column (LiChrospher 100 RP18 EC-5, 125 × 4 mm, CS-Chromatographie, Langerwehe, Germany) with methanol/water (9:1) (A) and methanol (B) as the mobile phase. The following gradient was used at a flow rate of 1.5 mL/min: 0 min B—0%; 10 min B—100%; 32.5 min B—100%. The quantification of decaprenoxanthin was calculated based on a β-carotene standard (Merck, Darmstadt, Germany) and reported as β-carotene equivalents.

### 4.4. Analysis of Fluorescence via Flow Cytometry

Cell cultures were analyzed regarding their fluorescent intensity. Samples were diluted to a final OD_600_ of 0.1 with pure CGXII medium and immediately analyzed with the FACS Gallios^TM^ (Beckman Coulter GmbH, Krefeld, Germany). Alternatively, samples for fluorescence analysis were harvested and stored at 4 °C. *C. glutamicum* (pEPR1) was used as the autofluorescence reference. The GFPuv signal was measured with a blue solid-state laser at 405 nm excitation and fluorescence was detected using a 525/50 nm band-pass filter.

### 4.5. Overproduction and Purification of the Transcriptional Regulator CrtR

After transformation of the pET16b derivatives in *E. coli* BL21(DE3) or *E. coli* BL21(DE3) (pLysS) transformants carrying the respective plasmids pET16b-*crtR_Cg_*, pET16b-*crtR_Cc_*, pET16b-*crtR_Aj_*, pET16b-*crtR_Pn_*, pET16b-*crtR_Ml_* and pET16b-*crtR_Pc_* were grown at 37 °C in 500 mL LB medium with 10 µg/mL ampicillin to an OD_600_ of 0.5 before adding IPTG (0.5 mM) for induction of the gene expression. After induction, cells were cultivated at 21 °C for an additional 4 h and were harvested by centrifugation. Pellets were stored at −20 °C. Crude extract preparation and protein purification via Ni-NTA chromatography was performed as described elsewhere [23]. The purified regulator proteins were used for EMSA experiments without removing the N-terminal His-tag. 

### 4.6. Electrophoretic Mobility Shift Assay (EMSA)

To analyze the physical protein–DNA interaction between the different CrtR proteins and their putative native target DNA, bandshift assays were performed [77]. The His-tagged CrtR proteins were mixed in varying molar excess with 30–90 ng of PCR amplified and purified promoter fragments of the target genes in bandshift (BS) buffer (50 mM Tris–HCl, 10% (*v*/*v*) glycerol, 50 mM KCl, 10 mM MgCl_2_, 0.5 mM EDTA, pH 7.5) in a total volume of 20 µL. The 5′ UTR of *crtR* genes were PCR-amplified and purified with NucleoSpin kit (MACHEREY-NAGEL GmbH & Co. KG, Düren, Germany). Promoter fragments were amplified using the respective oligonucleotide pairs (Table 2). A 78 bp-fragment of the upstream region of cg2228 was added in every sample as a negative control using oligonucleotides cg2228_fw and cg2228_rv. After 30 min of incubation at room temperature, gel shift samples were separated on a native 6% (*w*/*v*) polyacrylamide. Additionally, the binding affinity in the presence of 100–650 µM GGPP as effector was analyzed by incubation of the protein with the effector under buffered conditions for 15 min at room temperature prior to the addition of the promoter. Subsequently, the gel shift samples were separated on a 6% DNA retardation gel (Life Technologies GmbH, Darmstadt, Germany) at 100 V buffered in 44.5 mM Tris, 44.5 mM boric acid and 1 mM EDTA at pH 8.3. Staining of the DNA was achieved with ethidium bromide.

### 4.7. Extraction of GGPP and LC-MS Analysis

For isolation of GGPP, 10 mL of culture were harvested at 4000 rpm and 15 min. The supernatant was removed and the cells stored till further use (−80 °C). The cell pellet was defrosted on ice and resuspended in 600 µL acidified methanol (pH 5). Pyrophosphates were extracted by 3 × 30 s shaking in silamat (Ivoclar Vivadent AG, Schaan, Liechtenstein) in the presence of 300 µL silica beads. The clear supernatant was used for LC-MS analysis after subsequent centrifugation for 10 min at 13,000× *g*. LC-MS measurement was performed on a LaChrom ULTRA system (San Jose, CA, USA) using a SeQuant Zic-pHILIC column (5 µm 150 × 2.1 mm) (Merck Millipore, Darmstadt, Germany). As a buffer system, 10 mM ammonium bicarbonat pH 9.3 (A) and acetonitrile (B) was used with a flow rate of 0.2 mL/min; 0–5 min 5% A (const.), 5–20 min 35% A (gradient), 20–25 min 5% A (gradient), 25–35 min 5% A (const.); pre-run 15 min. with 2 µL. Isoprenoid pyrophosphates were identified using a micrOTOFQ (Bruker Daltonics, Billerica, MA, USA) according to their masses (GPP 313.0601; FPP 381.1227; GGPP 449.1853 [M-H]^-^) and elution time in accordance to a standard (Sigma-Aldrich, Merck, Darmstadt, Germany).

## Figures and Tables

**Table 1:** Strains, genomic DNA and plasmids used in this study.

Strain, gDNA or Plasmid	Relevant Characteristics or Sequence	Reference
*E. coli* strains		
*E.coli* DH5α	F-*thi*-1 *endA1 hsdr17*(r-, m-) *supE44* Δ*lacU169* (Φ80*lacZ*ΔM15) *recA1 gyrA96*	[42]
S17-1	*recA pro hsdR* RP4-2-Tc::Mu-Km::Tn7 integrated into the chromosome	[43]
*E.coli* BL21 (DE3)	*F– ompT gal dcm lon hsdSB(rB–mB–) λ(DE3 [lacI lacUV5-T7p07 ind1 sam7 nin5]) [malB+]K-12(λS)*	[44]
*E.coli* BL21 (DE3) (pLysS)	*F– ompT gal dcm lon hsdSB(rB–mB–)* *λ* *(DE3 [lacI lacUV5-T7p07 ind1 sam7 nin5]) [malB+]K-12(* *λ* *S) pLysS[T7p20 orip15A](CmR)*	Promega
***C. glutamicum strains***		
*C. glutamicum* WT	ATCC 13032, wild type	[45]
WTΔ*crtR*	ATCC 13,032 with deletion of *crtR* (cg0725)	[23]
WTΔ*crtB*Δ*crtB2I’I2*	ATCC 13,032 with deletion of *crtB* (cg0721) and *crtB2I’I2* (OP_cg2672)	this work
GGPPA	WTΔ*crtB*Δ*crtB2I’I2* derivative with plasmid-driven IPTG-inducible expression of MEP pathway genes *dxs* (cg2083) and *idi* (cg2531) from pEKEx3 and the GGPP synthase gene *idsA* (cg2384) from pEC-XT.	this work
GGPPB	WTΔ*crtB*Δ*crtB2I’I2* derivative with plasmid-driven IPTG-inducible expression of MEP pathway genes *dxs* (cg2083) and *idi* (cg2531) from pEKEx3 and the GGPP synthase gene *idsA* (2384) and primary sigma factor gene *sigA* (cg2092) from pEC-XT.	this work
**Genomic DNA**		
*Acidipropionibacterium jensenii*	Wild type, DSM 20535, ATCC 4868	[46], DSMZ
*Corynebacterium callunae*	Wild type, DSM 20147, ATCC 15991	[47]
*Micrococcus luteus*	Wild type, DSM 20030, ATCC 4698	[48], DSMZ
*Paenarthrobacter nicotinovorans*	Wild type, DSM 420, ATCC 49919	[49], DSMZ
*Pseudarthrobacter chlorophenolicus*	Wild type, DSM 12829, ATCC 700700	[50], DSMZ
**Plasmids**		
pEPR1	Km^R^, pCG1 *oriV_CG_*, *gfp*uv, promoterless, *C. glutamicum/E.coli* shuttle promoter-probe vector	[37]
pEPR1_P*crtE*	pEPR1 derivate containing the promoter of *crtE* (P*crtE*)	[23]
pTEST	pEPR1_P*crtE* derivate containing an additional expression cassette for expression of *crtR* orthologs from the *gap* promoter	this work
pTEST_*crtR_Cg_*	pTEST derivate for expression of the *crtR* from *C. glutamicum*	this work
pTEST_*crtR_Cc_*	pTEST derivate for heterologous expression of the *crtR* orthologs from *C. callunae*	this work
pTEST_*crtR_Aj_*	pTEST derivate for heterologous expression of the *crtR* ortholog from *A. jensenii*	this work
pTEST_*crtR_Pn_*	pTEST derivate for heterologous expression of the *crtR* ortholog from *P. nicotinovorans*	this work
pTEST_*crtR_Pc_*	pTEST derivate for heterologous expression of the *crtR* ortholog from *P. chlorophenolicus*	this work
pTEST_*crtR_Ml_*	pTEST derivate for heterologous expression of the *crtR* ortholog from *M. luteus*	this work
pET16b	Expression plasmid for production of His-tagged proteins	Novagen
pET16b_*crtR_Cg_*	pET16b derivate for production of His-tagged CrtR from *C. glutamicum*	this work
pET16b_*crtR_Cc_*	pET16b derivate for production of His-tagged CrtR *C. callunae*	this work
pET16b_*crtR_Aj_*	pET16b derivate for production of His-tagged CrtR *A. jensenii*	this work
pET16b_*crtR_Pn_*	pET16b derivate for expression of the *crtR* from *P. nicotinovorans*	this work
pET16b_*crtR_Pc_*	pET16b derivate for production of His-tagged CrtR *P. chlorophenolicus*	this work
pET16b_*crtR_Ml_*	pET16b derivate for production of His-tagged CrtR *M. luteus*	this work
pEKEx3	Spec^R^, P*tac lacI^q^*, pBL1 *oriVCg*, *C. glutamicum/E. coli* expression shuttle vector	[51]
pEKEx3_*dxs_idi*	pEKEx3 derivate for IPTG-inducible expression of *dxs* and *idi* from *C. glutamicum* containing an artificial ribosome binding site	this work
pEC-XT99A	Tet^R^, P*trc lacI^q^*, pGA1 *oriVCg*, *C. glutamicum/E. coli* expression shuttle vector	[52]
pEC-XT_*idsA*	pEC-XT99A derivate for IPTG-inducible expression of *idsA* from *C. glutamicum* containing an artificial ribosome binding site	this work
pEC-XT_*idsA_sigA*	pEC-XT99A derivate for IPTG-inducible expression of *idsA* and *sigA* from *C. glutamicum* containing an artificial ribosome binding site	this work

**Table 2:** Oligonucleotides used in this study.

Oligonucleotide (5′→3′)	
NH45	CATGCCTGCAGGTCGACTCTAGAGGAAAGGAGGCCCTTCAGATGGGAATTCTGAACAGTATTTCAA
NH46	GTTCGTGTGGCAGTTTTATTCCCCGAACAGGGAATC
NH47	AACTGCCACACGAACGAAAGGAGGCCCTTCAGATGTCTAAGCTTAGGGGCATG
NH48	ATTCGAGCTCGGTACCCGGGGATCTTACTCTGCGTCAAACGCTTC
NH49	ATGGAATTCGAGCTCGGTACCCGGGGAAAGGAGGCCCTTCAGATGGCTTACTCCGCTATGGCTA
NH50	GCATGCCTGCAGGTCGACTCTAGAGGATCTTAGTTCTGGCGGAAAGCAA
NH51	GTTCGTGTGGCAGTTTTAGTTCTGGCGGAAAGCAA
NH52	ATGGAATTCGAGCTCGGTACCCGGGGAAAGGAGGCCCTTCAGATGGACTTTCCGCAGCAACTCG
NH53	GCATGCCTGCAGGTCGACTCTAGAGGATCTTATTTATTACGCTGGATGATGTAGTCC
NH54	GTTCGTGTGGCAGTTTTATTTATTACGCTGGATGATGTAGTCC
NH55	ATGGAATTCGAGCTCGGTACCCGGGGAAAGGAGGCCCTTCAGATGAGCAGTTTCGATGCCCA
NH56	GCATGCCTGCAGGTCGACTCTAGAGGATCTTACATCCGACGTTCGGTTGA
NH57	GTTCGTGTGGCAGTTTTACATCCGACGTTCGGTTGA
NH58	ATGGAATTCGAGCTCGGTACCCGGGGAAAGGAGGCCCTTCAGATGGTAGAAAACAACGTAGCAA
NH59	GCATGCCTGCAGGTCGACTCTAGAGGATCTTAGTCCAGGTAGTCGCGAAG
NH60	AACTGCCACACGAACGAAAGGAGGCCCTTCAGATGGTAGAAAACAACGTAGCAA
NH63	GCAAAGTTGTTGTCGTAGTC
NH64	ATGAAAACGTTGTTGCCAT
NH65	ATGAAGACGCCACTGAC
NH66	CGGTGAGCTCGGCATCT
NH67	GTGCCTTGCGAGCTGTCT
TH17	CTGTTGATGACGACGAGGAG
pE-CXT fw	AATACGCAAACCGCCTCTCC
pE-CXT rv	TACTGCCGCCAGGCAAATTC
crtE-E	GTGACCATGAGGGCGAAAGC
crtE-F	TCACATAGTCCGGCGTTTGC
idsA-E	GCAGCTTCGCCAGAGTGTAT
idsA-F	CAATGCGGACAATGCTCCAG
581	CATCATAACGGTTCTGGC
582	ATCTTCTCTCATCCGCCA
P*gap* fw	TGGCCTTTTGCTGGCCTTTTGCTCACTGCGAAATCTTTGTTTCCCCG
P*gap* rv	GGATCCGTTGTGTCTCCTCTAAAGATT
*term* fw	AATCTTTAGAGGAGACACAACGGATCCTTTTGGCGGATGAGAGAA
*term* rv	AATCAGGGGATAACGCAGGAAAGAACAAAAGAGTTTGTAGAA
NA25- Cg fw	TACAATCTTTAGAGGAGACACAACGGAAAGGAGGCCCTTCAGATGCTGAATATGCAGGAACCA
NA26- Cg rv	AAAATCTTCTCTCATCCGCCAAAAGTTACTCCGTGTTGAGCCATGG
NA27- Cc fw	TACAATCTTTAGAGGAGACACAACGGAAAGGAGGCCCTTCAGATGTCCGATCCGCAAGAACC
NA28- Cc rv	AAAATCTTCTCTCATCCGCCAAAAGTTAATGTGAGGAAGACTCGAAC
NA31- Aj fw	TACAATCTTTAGAGGAGACACAACGGAAAGGAGGCCCTTCAGATGAGTGAAGACCGCGATG
NA32- Aj rv	AAAATCTTCTCTCATCCGCCAAAAGTTACCGCGGGTGGCGC
NA33-An fw	TACAATCTTTAGAGGAGACACAACGGAAAGGAGGCCCTTCAGATGTCCAGTCTTGAAGAAATGC
NA34-An rv	AAAATCTTCTCTCATCCGCCAAAAGTTAGCGTGGAGCCGCAG
NA39- Ml fw	TACAATCTTTAGAGGAGACACAACGGAAAGGAGGCCCTTCAGATGACCACGCAGCCCC
NA40- Ml rv	AAAATCTTCTCTCATCCGCCAAAAGTTACGGGTCCTCCGGGG
NA41- Pc fw	TACAATCTTTAGAGGAGACACAACGGAAAGGAGGCCCTTCAGATGAACGGCAACAATCCG
NA42- Pc rv	AAAATCTTCTCTCATCCGCCAAAAGTTACCCGGCTGGACGC
HN83-Cg-fw	GCGGCCATATCGAAGGTCGTCATCTGAATATGCAGGAACCAG
HN84-Cg-rv	TAGCAGCCGGATCCTCGAGCATTACTCCGTGTTGAGCCATG
HN85-Cc-fw	GCGGCCATATCGAAGGTCGTCATTCCGATCCGCAAGAACCCC
HN86-Cc-rv	TAGCAGCCGGATCCTCGAGCATTAATGTGAGGAAGACTCGAAC
HN87-Aj-fw	GCGGCCATATCGAAGGTCGTCATAGTGAAGACCGCGATGC
HN88-Aj-rv	TAGCAGCCGGATCCTCGAGCATTACCGCGGGTGGCGC
HN89-Pn-fw	GCGGCCATATCGAAGGTCGTCATTCCAGTCTTGAAGAAATGCC
HN90-Pn-rv	TAGCAGCCGGATCCTCGAGCATTAGCGTGGAGCCGCAG
HN93-Ml-fw	GCGGCCATATCGAAGGTCGTCATACCACGCAGCCCCCC
HN94-Ml-rv	TAGCAGCCGGATCCTCGAGCATTACGGGTCCTCCGGGG
HN95-Pc-fw	GCGGCCATATCGAAGGTCGTCATAACGGCAACAATCCGGGC
HN96-Pc-rv	TAGCAGCCGGATCCTCGAGCATTACCCGGCTGGACGC
Pc-PcrtR-fw	TGCCTTCCATGCGGATGGTC
Pc-PcrtR-rv	TGCCCGGATTGTTGCCGTTC

## Supplementary Materials

The following figures are available online at https://www.mdpi.com/1422-0067/21/15/5482/s1. Figure S1: Biosensor plasmids, Figure S2: Validation of the pTEST vector system for promoter activity assay and expression of crtR orthologs.
