# Supplementary material for: Corynebacterium glutamicum CrtR and Its Orthologs in Actinobacteria: Conserved Function and Application as Genetically Encoded Biosensor for Detection of Geranylgeranyl Pyrophosphate
Source: Int J Mol Sci. 2020 Jul 31;21(15):5482. doi: 10.3390/ijms21155482 (PMC7432914; doi:10.3390/ijms21155482)
Supplement: Supplementary file 1 [file ijms-21-05482-s001.pdf]

Supplementary Materials:

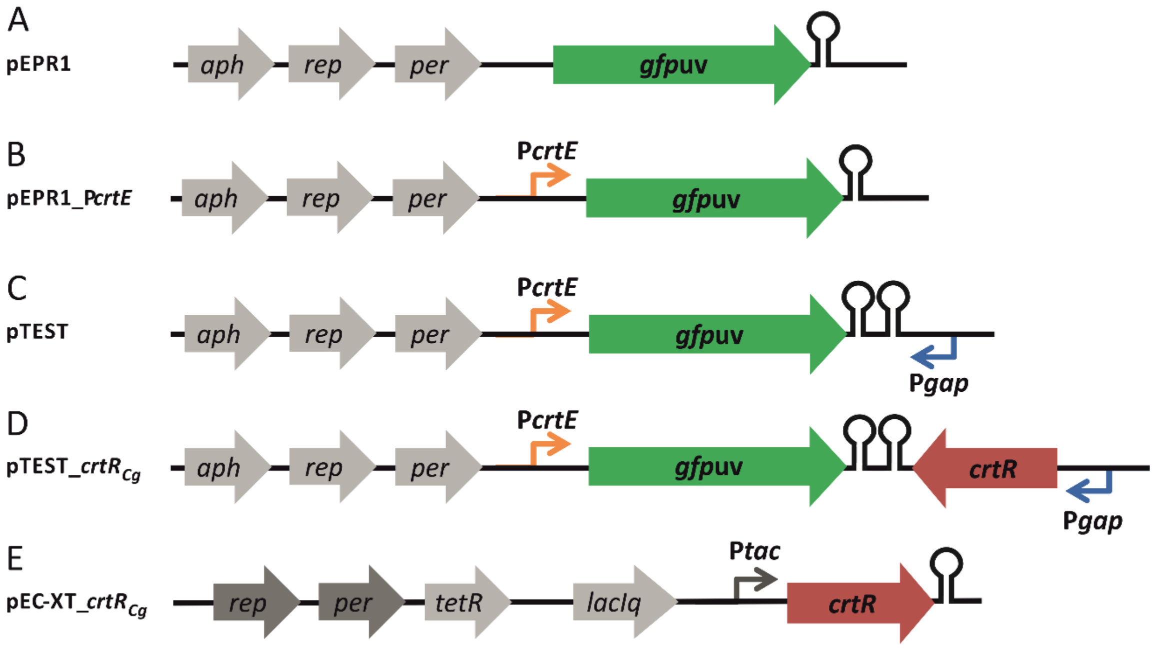

Figure S1. Biosensor plasmids. The promoter probe vectors pEPR1 (A) and pEPR1_P*crtE* (B) and their derivatives pTEST (C) and pTEST_P*crtE* (D) are shown with *crtR* overexpression plasmid pEC-XT_*crtR*$_{Cg}$ (E). Genetic elements: P*crtE*: promoter of the *crt* operon; *gfp*uv: fluorescence reporter; P*gap*: promoter of *gapA*; *crtR*: gene for transcriptional regulator of P*crtE; aph*: kanamycin resistance gene; *tetR*: tetracyclin resistance gene; *rep*/*per*: origin of replication; *T*$_1$/*T*$_2$: terminators.

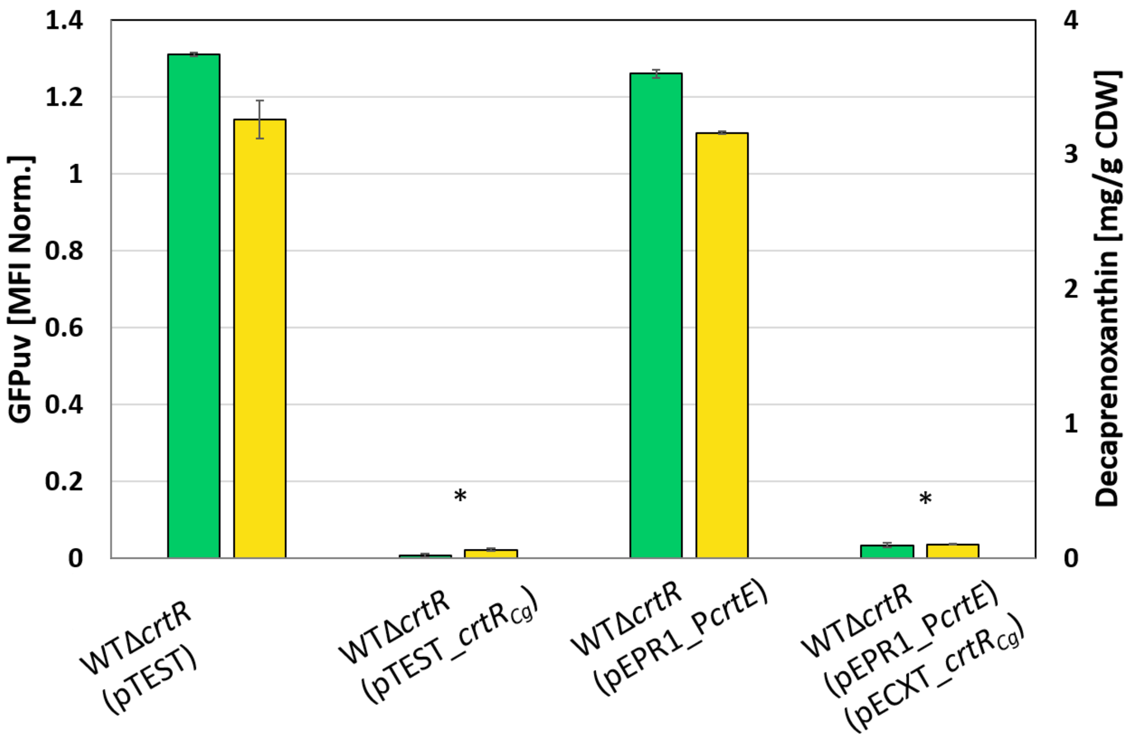

Figure S2. Validation of the pTEST vector system for promoter activity assay and expression of *crtR* orthologs. GFPuv signals (green) from cells grown in exponential growth phase were

normalized to autofluorescence and are given as mean fluorescence intensities. Decaprenoxanthin concentrations (orange) were determined in the stationary growth phase and were calculated based on a β-carotene standard.
